# Stakeholder perspectives on the costs and benefits of circular construction

**DOI:** 10.1038/s41598-024-81741-z

**Published:** 2024-12-03

**Authors:** Ferhat Karaca, Aidana Tleuken, Hamad Hassan Awan, Rand Askar, Mustafa Selçuk Çıdık, Anel Iskakova, Ali Turkyilmaz, Thomas Laudal, Serdar Durdyev, Huseyin Atakan Varol, Adriana Salles, Diāna Bajāre, Tatjana Tambovceva, Gabriel Zsembinszki, Genesis Camila Cervantes Puma, Zhanna Kapsalyamova, Dorina Kripa, Dina Azhgaliyeva, Xhesila Nano, Luisa F. Cabeza, Luís Bragança

**Affiliations:** 1 Department of Civil and Environmental Engineering, School of Engineering and Digital Sciences, Nazarbayev University, Astana, Kazakhstan; 2 Department of Civil Engineering, ISISE, ARISE, University of Minho, Guimarães, Portugal; 3 The Bartlett School of Sustainable Construction, University College London, London, UK; 4 Department of Innovation Management and Marketing, UiS School of Business and Law, University of Stavanger, Stavanger, Norway; 5 Department of Engineering and Architectural Studies, Ara Institute of Canterbury, Christchurch, New Zealand; 6 Institute of Smart Systems and Artificial Intelligence (ISSAI), Nazarbayev University, Astana, Kazakhstan; 7 Institute of Sustainable Building Material and Engineering Systems, Faculty of Civil Engineering and Mechanical Engineering, Riga Technical University, Riga, Latvia; 8 Governance and Security Institute, Faculty of Engineering Economics and Management, Riga Technical University, Kalnciema Str. 6-506, Riga, Latvia; 9 GREiA Research Group, University of Lleida, 25001 Lleida, Spain; 10 Department of Economics, School of Sciences and Humanities, Nazarbayev University, Astana, Kazakhstan; 11 University of Tirana, Tirana, Albania; 12 Asian Development Bank Institute (ADBI), Tokyo, Japan; 13 Department Industrial Engineering and Business Information Systems, University of Twente, Enschede, The Netherlands

**Keywords:** Construction industry, Circular economy, Recycling, Waste management, Reuse, Machine learning, Civil engineering, Climate change

## Abstract

The construction industry significantly impacts the environment through natural resource depletion and energy consumption, leading to environmental degradation. Circular Economy (CE) material efficiency strategies—such as material reuse, design for disassembly, prefabrication, and recycling—offer promising solutions for reducing resource consumption and waste. This paper explores stakeholders’ perspectives on the costs and benefits of implementing CE material efficiency strategies in the construction industry, using the 3-R (Reduce, Reuse, Recycle) framework. By analyzing data from 382 participants, it assesses perceptions of costs and benefits, uncovering regional differences. The findings highlight that studied European stakeholders prioritize reuse and design for disassembly, while studied non-European countries focus on offsite production and material reuse optimization. Despite these differences, both groups view waste reduction as a key benefit, due to the cost savings it provides. By highlighting regional drivers and barriers to CE adoption, this research establishes a foundation for developing targeted policies and collaborative strategies to advance CE implementation in construction worldwide.

**Supplementary Information:**

The online version contains supplementary material available at 10.1038/s41598-024-81741-z.

## Introduction

The construction industry, heavily reliant on natural resources and characterized by high energy consumption, contributes significantly to environmental issues such as elevated greenhouse gas (GHG) emissions, air pollution, environmental degradation, and global warming. Specifically, building construction alone accounts for 10% of global GHG emissions and 6% of global energy consumption^1^. Reducing emissions within the construction sector is crucial to meet global net-zero emission targets. Recognizing this, adopting CE principles—aligned with Industry 4.0 objectives—holds significant promise for reducing GHG emissions across the construction supply chain.

Circular Economy (CE) is a systemic approach aimed at decoupling economic development and activity from the consumption of finite resources^2^. In response to the inefficiencies of the current linear economic model, CE strives to establish a closed-loop system within the value chain, maximizing resource use and minimizing waste generation^3^. Notably, approximately 80% of construction materials end up as waste at the end of their useful life^4,5^. When comparing different approaches to building construction, CE material efficiency strategies present diverse methods for using construction materials more sustainably, which includes designing buildings for adaptability, disassembly, reuse, and recycling. Examples include dry connecting methods such as bolting, allowing for easy maintenance and disassembly, and keeping structural elements visible for simplified inspection^6,7^. Prefabrication, where components are prepared offsite, also facilitates easy disassembly later and minimizes onsite work^8^. Building material reuse and recycling present further opportunities to reduce construction and demolition waste (C&DW), achievable through closed-loop and open-loop methods. This involves reusing and recycling materials into the same or different products, creating a regenerative model^8^. Additionally, circular procurement, a forward-looking practice supporting the CE transition, emphasizes sustainable material usage and extends to the servitization of the construction industry’s offerings^9^. Through such efforts, CE principles enable a shift towards circularity in the industry, emphasizing long-term resource management and sustainability.

However, despite the available literature on circular construction technologies and solutions, these options are often high-cost. Studies suggest that, although CE holds potential positive impacts for the environment and society, construction organizations—especially Small and Medium Enterprises (SMEs)—perceive it as economically challenging to implement^11^. Consequently, understanding stakeholder perceptions of the costs and benefits of adopting CE material efficiency strategies is essential. By investigating these perspectives, this study aims to uncover perceived barriers and motivators for CE within the construction industry, providing insight into the development of effective, evidence-based policies that can encourage adoption of CE material efficiency strategies across the sector and contribute to broader environmental and economic sustainability goals.

This study aims to examine stakeholder perceptions of the costs and benefits specifically associated with material-focused CE strategies in the construction industry to provide evidence-based insights that inform policy development. By addressing these perspectives, the research aims to encourage a shift toward more sustainable practices across the construction sector, contributing to broader environmental and economic sustainability goals.

The research focuses on the following objectives:

- To what extent do CE material efficiency strategies influence the overall costs and benefits for organizations in the construction industry?
- What are the primary cost drivers and benefits of implementing CE material efficiency strategies?

## Literature review: costs and benefits of CE material efficiency strategies

The construction industry, primarily driven by financial considerations, often resists innovations due to uncertainties surrounding associated costs and benefits^7^. According to^10^, the primary drivers of a sustainable construction sector include government regulations, internal and external stakeholder pressures, and perceptions of cost-effectiveness. Previous studies emphasize the need for a clear understanding of the costs and benefits of circular business models in construction to encourage decision-makers to adopt circularity practices. Notably, research underscores that although CE is acknowledged for its potential positive impact on the environment and society, uncertainties persist around its costs and benefits for the firms in the construction industry, especially for the SMEs^7,10,11^. Financial institutions, often lacking experience with circular models, are also more likely to classify circular business practices as high-risk.

Adopting CE material efficiency strategies, such as material reuse and recycling, involves substantial initial investment, including the purchase of recycling and sorting equipment, waste processing plants, and relevant technologies^12,13^. Four main factors drive the costs associated with CE in construction: market development, measurement methods, policy, and knowledge. Geographical factors also play a role; in areas with low energy costs and established cement industries, additional investment in concrete recycling machinery may be deprioritized as energy savings are not prioritized in cost-benefit analyses. Environment-friendly and recycled materials generally carry a higher price tag than traditional ones^12^. Additionally, assessing the quality and usability of reclaimed materials for different applications can present significant challenges^14^. Enabling CE operations incurs various direct costs, including those associated with energy and water use for aggregate cleaning, transportation, and additional machinery and equipment maintenance^15^. The CE approach also requires a shift in product design methods, such as Design for Disassembly (DfD) and modular design, involving changes in technological software and specialized skills compared to those of the linear economy^8^. Therefore, construction professionals require training and education to acquire the skills needed for CE adoption. Beyond financial considerations, aesthetic issues such as the visual impact of stockpiles, dust, noise, and odors from waste piles and processing facilities also need to be addressed^15^. Overcoming these complex challenges will support the construction industry in successfully adopting CE practices, thereby ensuring sustainable implementation.

The benefits of adopting CE material efficiency strategies in construction can be significant, ranging from waste reduction and decreased use of virgin resources to a lower environmental footprint associated with the production of new materials, including decreased energy consumption and reduced GHG emissions during transportation^15,16^. Material reuse through resale has been shown to be more advantageous than purchasing new materials, and refurbishment offers a cost-effective alternative to new construction^15^. Furthermore, adopting CE principles can enhance economic competitiveness by creating new markets, reducing dependency on imports, and generating job opportunities^8^. Adopting CE practices may also yield tax benefits since negative environmental impacts are currently subject to taxation^12^. Government and municipal regulations frequently act as primary drivers for CE adoption, particularly among construction SMEs, sometimes even outweighing purely financial motives^11^. Overall, adopting CE principles in the construction industry supports a shift toward a more sustainable, resource-efficient sector, and can generate both economic and the environmental benefits.

## Methodology

This paper evaluates stakeholder perspectives on the costs and benefits of implementing CE material efficiency strategies in construction by using a survey to gather insights from various stakeholders along the value chain. Employing non-probabilistic, convenience sampling, the survey reached a wide audience through social media, professional networks, and email lists, ensuring diverse representation by geography, age, and profession. Invitations were sent to a broad group, including contractors, suppliers, regulators, engineers, and architects, resulting in a robust collection of opinions on CE in construction. The survey provided qualitative data on the costs and benefits of CE material efficiency strategies as perceived by stakeholders. This data was analyzed using a supervised ensemble machine learning (ML) method, generating construction-specific models of cost and benefit perceptions. This approach offers a detailed view of stakeholder perspectives on the relevance and impact of CE material efficiency strategies within the construction industry.

### Development of the survey

This study focuses specifically on CE material efficiency strategies within the construction industry. To facilitate stakeholder engagement in an online survey, a curated list of high-priority, impactful strategies from the literature was selected, along with their respective potential costs and benefits^6–9,11,12,15,18^. The focus on CE material efficiency strategies streamlines the survey for participants, allowing it to capture relevant insights without overwhelming respondents with an extensive list. The selected CE material efficiency strategies, along with their respective costs and benefits, are detailed in Figures S1 and S2 in Supplementary Information II.

The identified CE material efficiency strategies were integrated into the survey questionnaire, which included questions about stakeholders’ opinions on the overall costs and benefits for organizations, as well as on specific primary cost drivers and benefits. The questionnaire began with general questions about the respondent’s country, experience, company size, and stakeholder type. This section was followed by Likert-scale (1–5) questions assessing respondents’ views on CE material efficiency strategies and their potential costs and benefits.

Following a thorough validation process, the survey questions were reviewed in two roundtable sessions conducted by the authors on March 29, 2023, and May 5, 2023. During the initial roundtable, each question was assessed individually to ensure comprehensive coverage of the topic. Subsequently, a pilot study was conducted with 25 academic and construction industry experts to gather feedback and identify any challenges. These experts, selected as pilot respondents, provided insights on survey aspects, including navigation, wording, content, and relevance. This process tested the survey questions to ensure readability and usability. Based on their feedback, a second roundtable discussion was held to refine and enhance the survey. As the focus of this paper is on circular material use, only the questions that were exclusively designed to gather information on this topic are used in the analysis. A sample of the questionnaire is provided in Supplementary Information I.

### Conducting the survey

The respondents were recruited through an online survey accessible online from June 1 to August 30, 2023. Nazarbayev University Institutional Research Ethics Committee reviewed ethical considerations for the survey questions and granted approval for all experimental protocols. All methods were carried out in accordance with relevant guidelines and regulations and informed consent was obtained from all respondents, in accordance with Nazarbayev University Institutional Research Ethics Committee approval (716/11052023). The survey supported diversity by offering versions in English, Turkish, Russian, Spanish, Norwegian, and Portuguese, based on requests from the authors’ countries. Translations were verified by bilingual native speakers to ensure reliability. Responses with more than 70% empty fields or entirely blank were excluded to uphold data analysis quality.

**Fig. 1:**
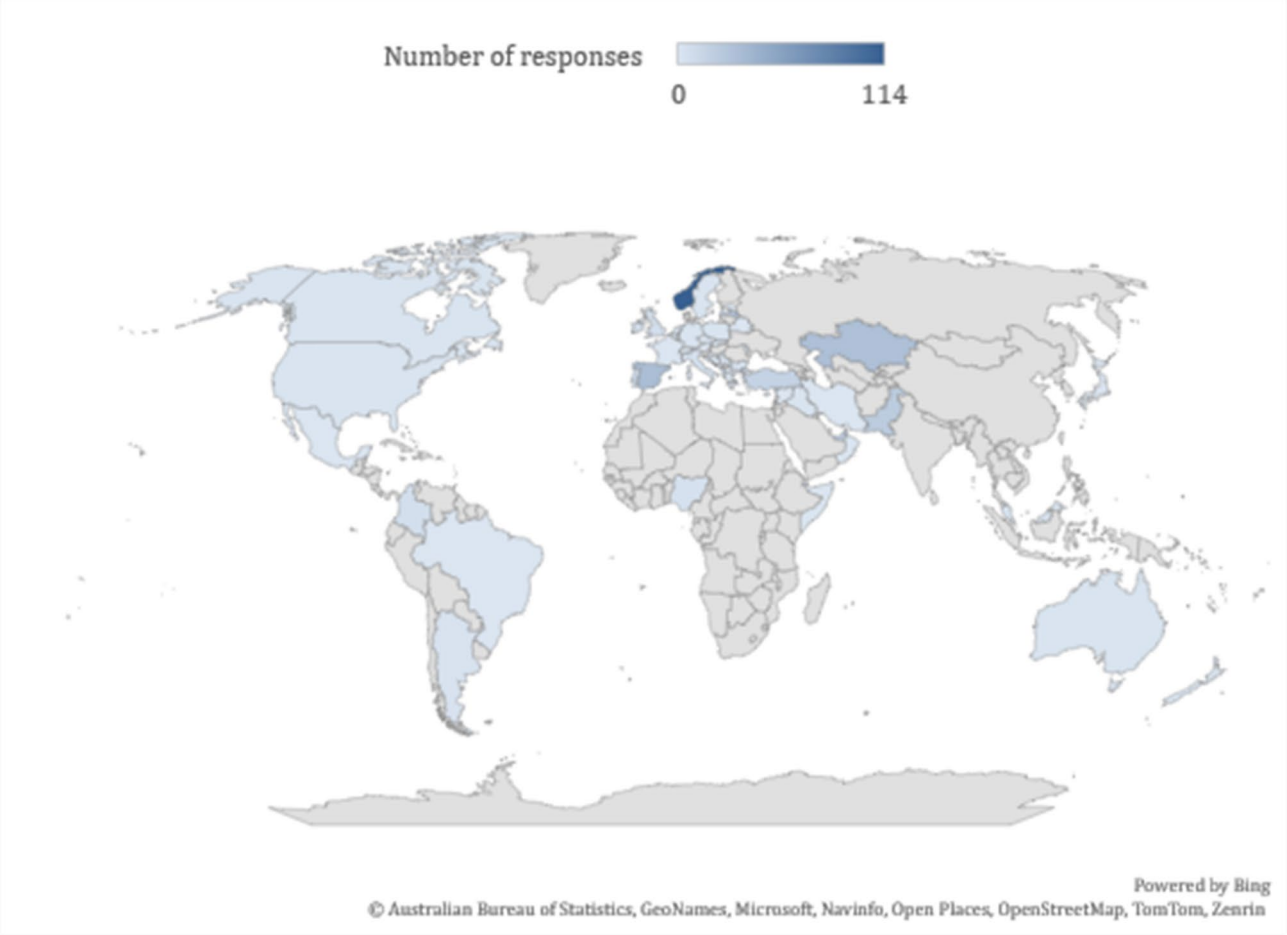
The overview of the composition of the respondents’ sample. Source: authors. The map was created using Microsoft Excel (version 2406) and utilizes Bing Maps for geographical data visualization. URL link to software: https://www.microsoft.com/en-us/microsoft-365/excel

The survey obtained 382 valid responses from individuals of different backgrounds and countries. Table 1 shows the countries and stakeholders which have passed the minimum requirements (number of respondents, *n* > 15). Norway was the most active in this survey, with 114 respondents. Figure 1 shows the overview of the countries of origin of the respondents.

**Table 1:** Stakeholders VS countries statistics. Source: Authors.

	KZ	LV	NOR	PAK	TR	SP	UAE	Other	Grand total
Academician/Researcher	5	3	3	7	9	16	0	57	100
Project manager	7	8	34	3	3	4	5	14	78
Designer/Architect/Engineer/Technician	9	3	26	3	4	12	3	16	76
Contractor	4	2	14	3	0	1	10	7	41
Manufacturer/Material Supplier	1	8	15	2	0	1	0	5	32
Client and/or Investor	2	2	3	4	0	0	3	5	19
Other	4	4	19	0	0	0	0	9	36
Total	32	30	114	22	16	34	21	113	382

KZ: Kazakhstan, LV: Latvia, NOR: Norway; PAK: Pakistan; TR: Turkiye, SP: Spain; UAE: United Arab Emirates.

### Data analysis

Machine Learning (ML) is an evolving field that uses examples and historical data to train machines in recognizing desired input-output patterns, thus gaining experience and improving responses^17^. ML applications span diverse fields, including manufacturing, education, finance, policy, and construction. In survey research, ML plays a crucial role in adaptive design, data processing, nonresponse adjustments, and weighting^18^. These techniques help identify interactions and nonlinear relationships among variables and are effective at classifying cases based on collected variables^18^.

**Fig. 2:**
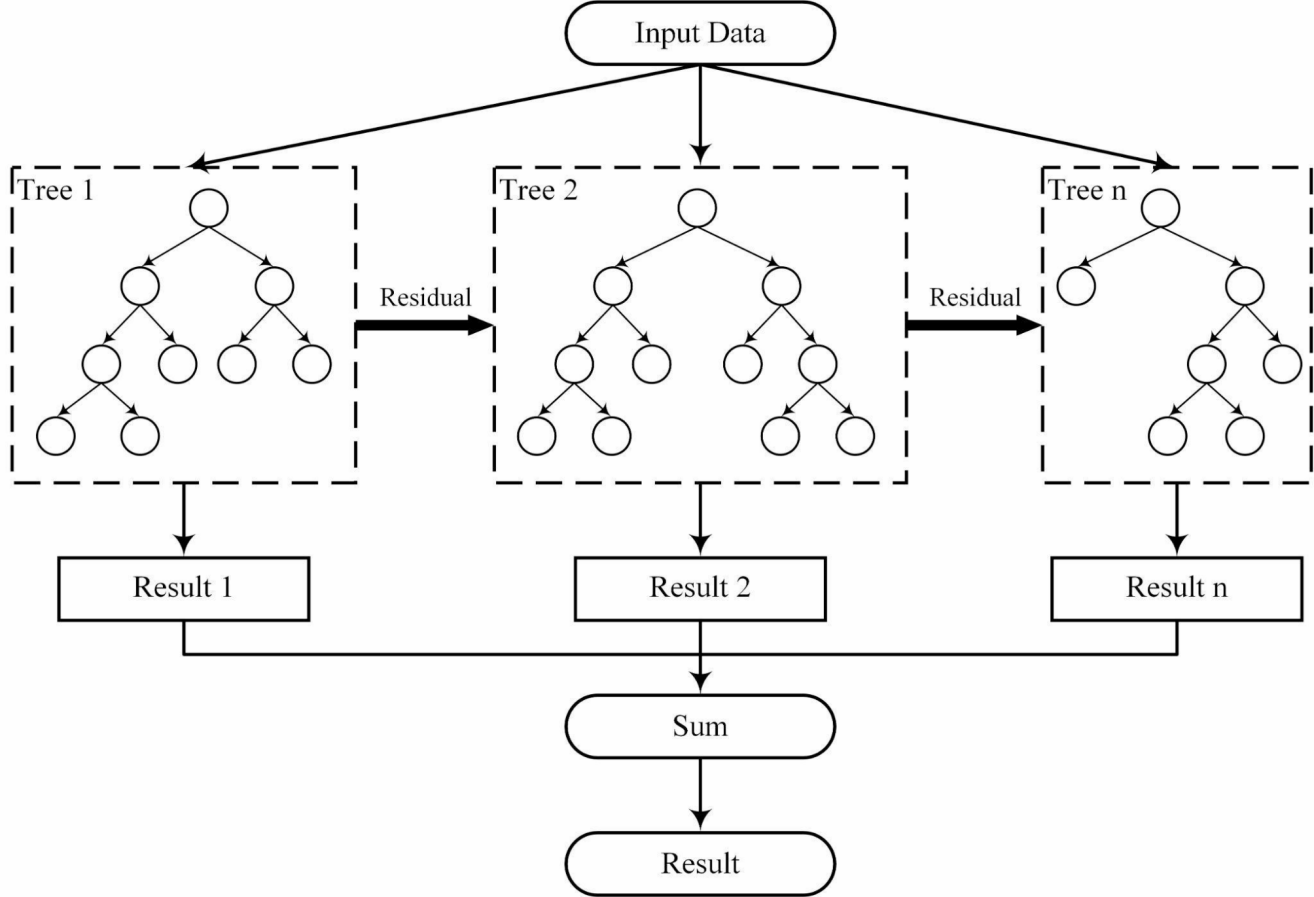
Dummy example of simple trees in XGBoost algorithm for this study. Source: Authors.

This study uses the XGBoost (eXtreme Gradient Boosted decision tree) algorithm and SHAP (SHapley Additive exPlanations) methods, which are popular among data scientists for regression and classification tasks^20,21,25–27^. XGBoost combines Gradient Descent and Boosting to create a robust model from simple decision trees, identifying hierarchical data relationships (see Fig. 2). These trees reveal dependencies among survey responses, offering insights into respondent behavior by showing how different responses influence each other. This approach improves estimator efficiency by reducing bias and stabilizing variance across numerous boosting rounds^22–24^.

SHAP analysis further enhances understanding by revealing the significance and influence of different features on predictions. For instance, in traffic studies, SHAP has shown that attributes related to speed significantly impact the likelihood of accidents. In this study, SHAP analysis highlights factors affecting the perceived costs and benefits of CE material efficiency strategies in construction, offering insights into respondent behaviors and factors influencing their views on CE.

The complexity of factors and opinions in the construction sector necessitates advanced data analysis techniques for survey data. XGBoost uncovers connections among survey questions, while SHAP interprets these findings by emphasizing the significance of responses. This approach provides insights into respondent opinions and preferences, making XGBoost an efficient and robust tool for this research.

In this study, XGBoost’s accuracy is assessed using the Mean Absolute Percentage Error (MAPE), with a threshold of 50% considered accurate [58]. An 80–20 split is used for the training and testing sets. Two models were created for each research question to differentiate between European and non-European countries, accounting for regional differences. Results derived from models based on the entire dataset are provided in Supplementary Information III.

Research using ML to improve cost-benefit models, especially for material costs, is limited^19^. This study seeks to fill this gap by using SHAP analysis and XGBoost to uncover correlations among survey questions. This approach enhances understanding of the internal relationships within the survey data, providing a comprehensive view of stakeholder perspectives on CE material efficiency strategies in the construction industry.

## Results and discussion

**Fig. 3:**
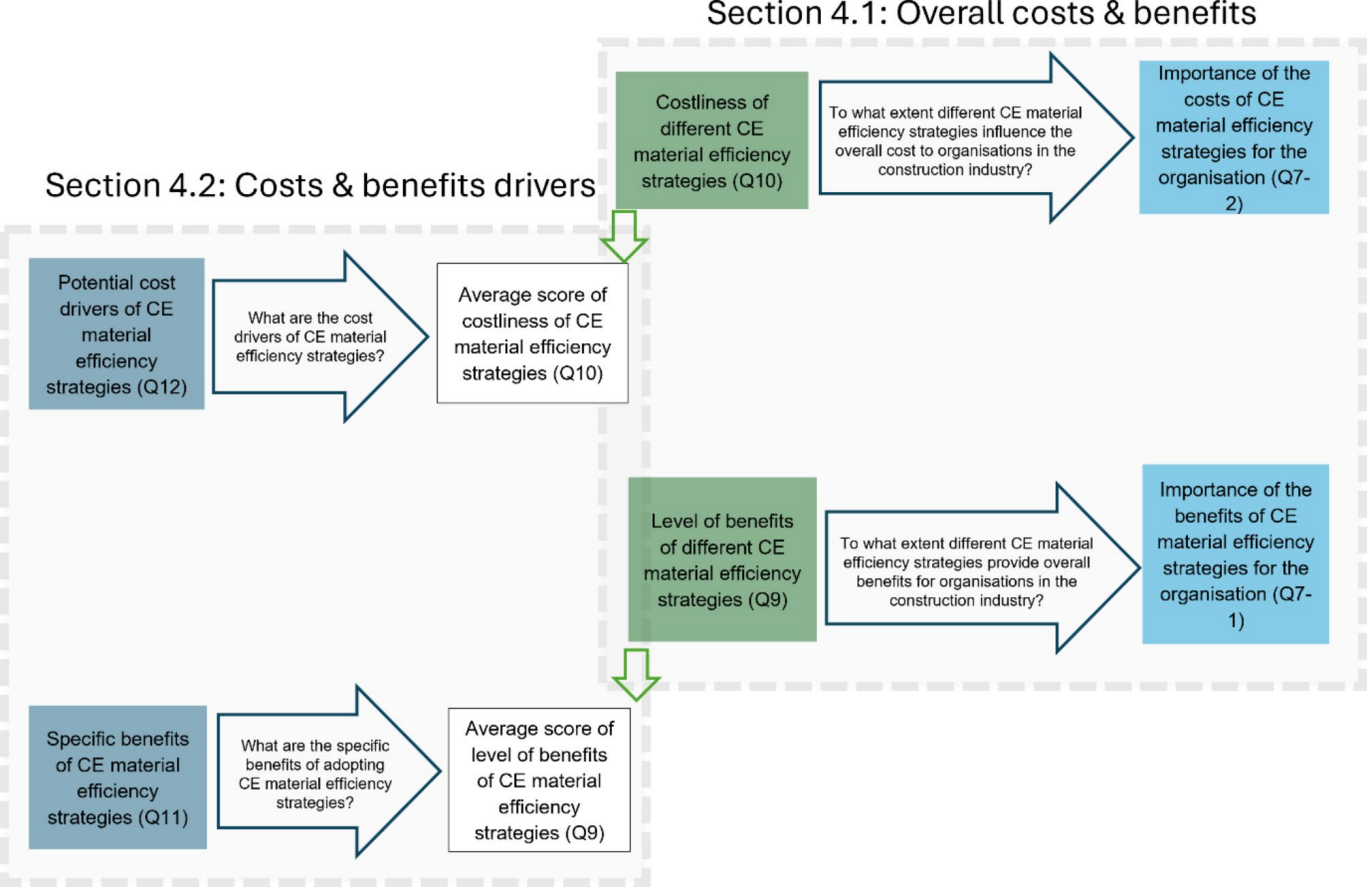
Research questions, survey construct, and inputs and outputs for the models. Source: Authors.

This section presents the results of the analysis on stakeholder perspectives regarding the costs and benefits of CE material efficiency strategies in construction. Each data presentation is immediately followed by a discussion to interpret and contextualize the findings. Our results on costs and benefits of adopting CE material efficiency strategies are divided into two main areas in accordance with research objectives: (1) contribution to overall organisational costs and benefits (section “Contribution of CE material efficiency strategies to overall costs and benefits”), (2) primary cost drivers and benefits (section “Cost drivers and benefits of CE material efficiency strategies”). Figure 3 demonstrates the inputs and outputs for the models in relation to the sections in Results and Discussions. For the full list of survey questions, see Supplementary Information I.

### Contribution of CE material efficiency strategies to overall costs and benefits

#### Costs

Several decision tree models were developed to address the research questions using specific survey elements. The first model focused on assessing the impact of CE material efficiency strategies on overall costs for construction organizations. This model was based on responses to Question 7.2, which asked, “How important are the costs of circular economy practices for your organization?” and on the perceived costliness of common CE material efficiency strategies, such as offsite production and material recycling (Questions 10.1–10.7).

**Fig. 4:**
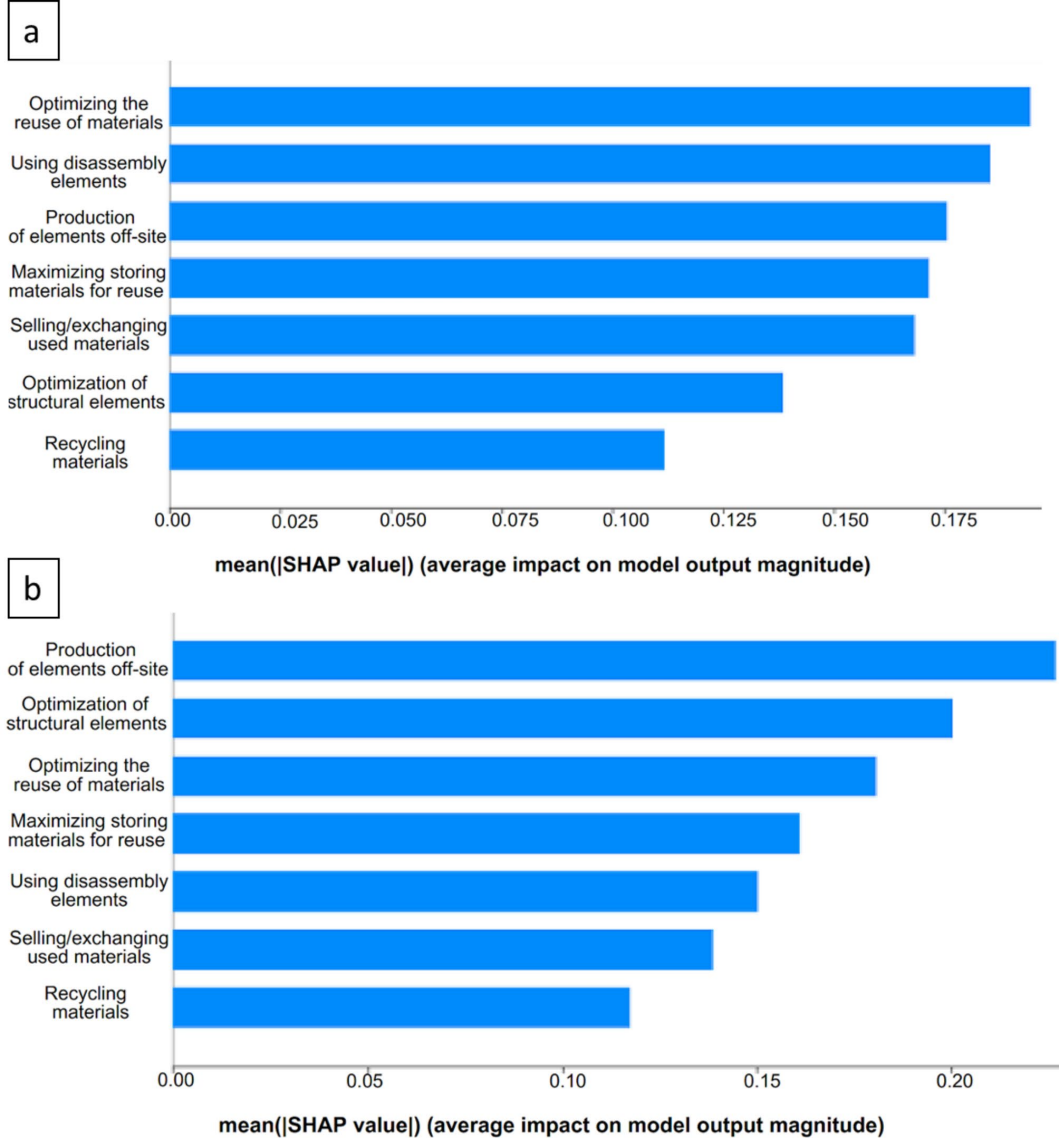
SHAP values plot from top to bottom (a) European, MAPE: 31.15%, (b) non-European, MAPE: 30.65%. Source: Authors.

The results are illustrated in Fig. 4a and b below. In the studied European countries, the top three factors that influence cost increases are perceived to be optimizing the reutilization of materials, using disassembly elements, and producing elements offsite. Conversely, optimizing structural elements and materials is deemed to contribute less to overall cost increases. Although some variation exists within the analyzed European cohort, the primary impacts on overall costs remain associated with the recovery of construction materials, disassembly requirements, and offsite production. Among the studied non-European countries, similar results appear, except that optimizing structural elements replaces ‘using disassembly elements’ as a top contributor to overall cost increases. Maximizing storage for reuse ranks as the fourth factor in both the studied European and non-European countries.

According to Fig. 4, cost reduction could be achieved in the studied European countries by focusing on the reutilization of elements, which stakeholders identify as the most significant contributor to overall costs among CE material efficiency strategies. Other notable influencing factors include design for disassembly (DfD) and offsite production of structural elements. In line with the European waste hierarchy, reuse takes priority over recycling^17^. Furthermore, recycling is perceived as contributing less to overall costs compared to reutilization (Fig. 4a), likely due to the established recycling practices for construction and demolition waste (C&DW)^18^ and the recognized value of C&DW materials.

The literature indicates that the primary challenge in reusing construction materials is the cost associated with these impacts, as confirmed by this analysis. Initial investments pose a barrier to entry in the studied European countries, as highlighted by respondents in Norway^19,20^. Latvian respondents note that reuse can be costly due to a lack of infrastructure^21^. Modular construction, an example of DfD and offsite production, also requires higher upfront investment, with no proven significant cost savings to date^22^. Nonetheless, Norway has one of the highest rates of offsite production in housing^22^. In Spain, sustainable waste management faces various influencing factors, including the need for recycling and re-evaluation by waste operators. Construction waste incurs direct costs for collection and recycling, as well as potential revenue through resale, supporting economic, social, and environmental sustainability^23,24^. The variation in results across the studied European countries may reflect different recovery and recycling models—centralized versus decentralized—that influence the impacts on overall costs^25^.

Among the studied non-European countries, concerns are similar (Fig. 4b), particularly regarding offsite production and optimizing reuse, which are key contributors to overall costs within CE material efficiency strategies. Unlike in the studied European countries, optimizing structural elements also ranks highly in terms of costs. In general, CE practices are less developed in the non-European regions studied. For example, CE is less widely studied outside Europe, apart from countries like the US and China, which were not represented in this study^26^. This may reflect lower emphasis on CE or limited representation in stakeholder networks associated with this research. In these countries (Kazakhstan, UAE, Pakistan, and Turkiye), the CE legislative framework is either new or absent^27,28^. In UAE, legislation emphasizes C&DW reuse and recycling, but stakeholders find compliance challenging^29^. Additionally, the lack of a centralized, non-competitive recycling model—though planned—may result in recycling being seen as a less significant contributor to overall costs, as associated costs are often overlooked^30,31^.

Global cost-benefit analyses of offsite production are essential for aiding decision-makers and identifying the most effective CE material efficiency strategies. Both European and non-European stakeholders recognize the impacts on overall costs associated with maximizing storage for reuse and implementing disassembly practices, highlighting an industry-wide challenge. Increased investment in R&D to make offsite production more affordable is vital, and collaborative platforms are needed to promote knowledge sharing among European stakeholders. This collaborative approach can foster a culture of learning and best practice sharing, advancing the cost-effective implementation of CE material efficiency strategies.

#### Benefits

**Fig. 5:**
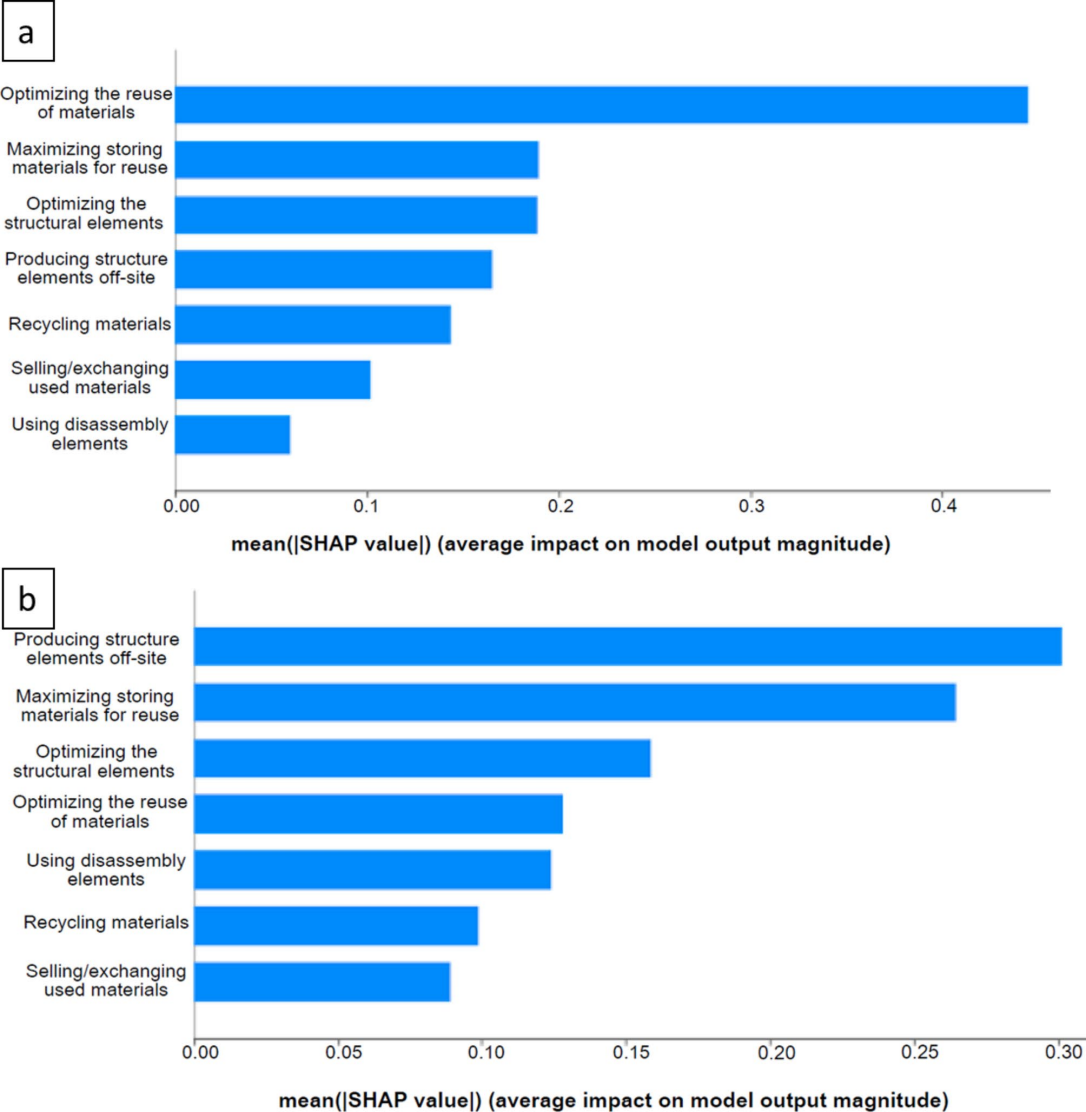
SHAP values plot from top to bottom (a) European, MAPE: 38.51%%, (b) non-European, MAPE: 27.48%. Source: Authors.

The second model assessed the importance of overall benefits for organisations associated with CE material efficiency strategies, linking responses to Question 7.1, which asked, “How important are the benefits of circular economy practices for your organization?” with levels of agreement on the benefits of these strategies (Question 9.1–9.7). Figure 5 provides the results for the studied (a) European and (b) non-European countries. The results reflect a strong perceived benefit of material reuse, optimization of structural elements, offsite production, and recycling in the studied European countries. The studied non-European countries also value offsite production, material reuse, and optimization of structural elements. Moreover, using structural elements that can be easily disassembled is also among the top factors perceived as providing benefits, suggesting the importance of developing construction methods where the end-of-life of a building is considered at the design stage.

The data presented in Fig. 5a shows the commitment of the studied European countries to promoting material efficiency to achieve circular economy objectives. This commitment indicates significant endorsement achieved through a combination of measures. One such measure is the optimization of the amount of material reuse, which involves identifying and prioritizing materials that can be reused and increasing the storage capacity for such materials. This approach has enabled construction companies to reduce their reliance on virgin materials, decreasing environmental impact and increasing resource efficiency. Another key strategy that the studied European stakeholders prioritize is reducing the number of structural elements used. This approach promotes efficiency by minimizing the use of materials and reducing waste. By adopting this approach, projects can significantly reduce their carbon footprint and enhance their sustainability credentials. The prioritization of material reuse in both overall cost and benefit models indicates a significant shift and dedication toward the circular economy approach. This is reinforced by legislation such as the Circular Economy Action Plan^32^, emphasizing the importance of reusing waste. These regulations not only encourage but also require innovative waste management strategies. Additionally, the commitment of the studied European participants to material reuse is further demonstrated by ongoing investments in recycling infrastructure and technology. According to a recent study^33^, Europe has devoted significant resources to improving infrastructure and introducing advanced recycling technologies, demonstrating its strong commitment to reusing materials. Recycling C&DW among European stakeholders is critical for bringing economic benefits, as this saves raw material supply costs, transportation, and disposal^34^.

For the studied non-European countries (Fig. 5b), priorities include offsite production, material reuse, and optimization of structural elements. The convergence on the importance of maximizing storage for reuse across both the studied European and non-European countries (Fig. 5a and b) implies a shared understanding of this practice’s economic and environmental benefits. The regional variations in prioritizing practices like reuse and recycling in Europe and disassembly in non-European countries show the importance of contextual factors.

### Cost drivers and benefits of CE material efficiency strategies

#### Costs

**Fig. 6:**
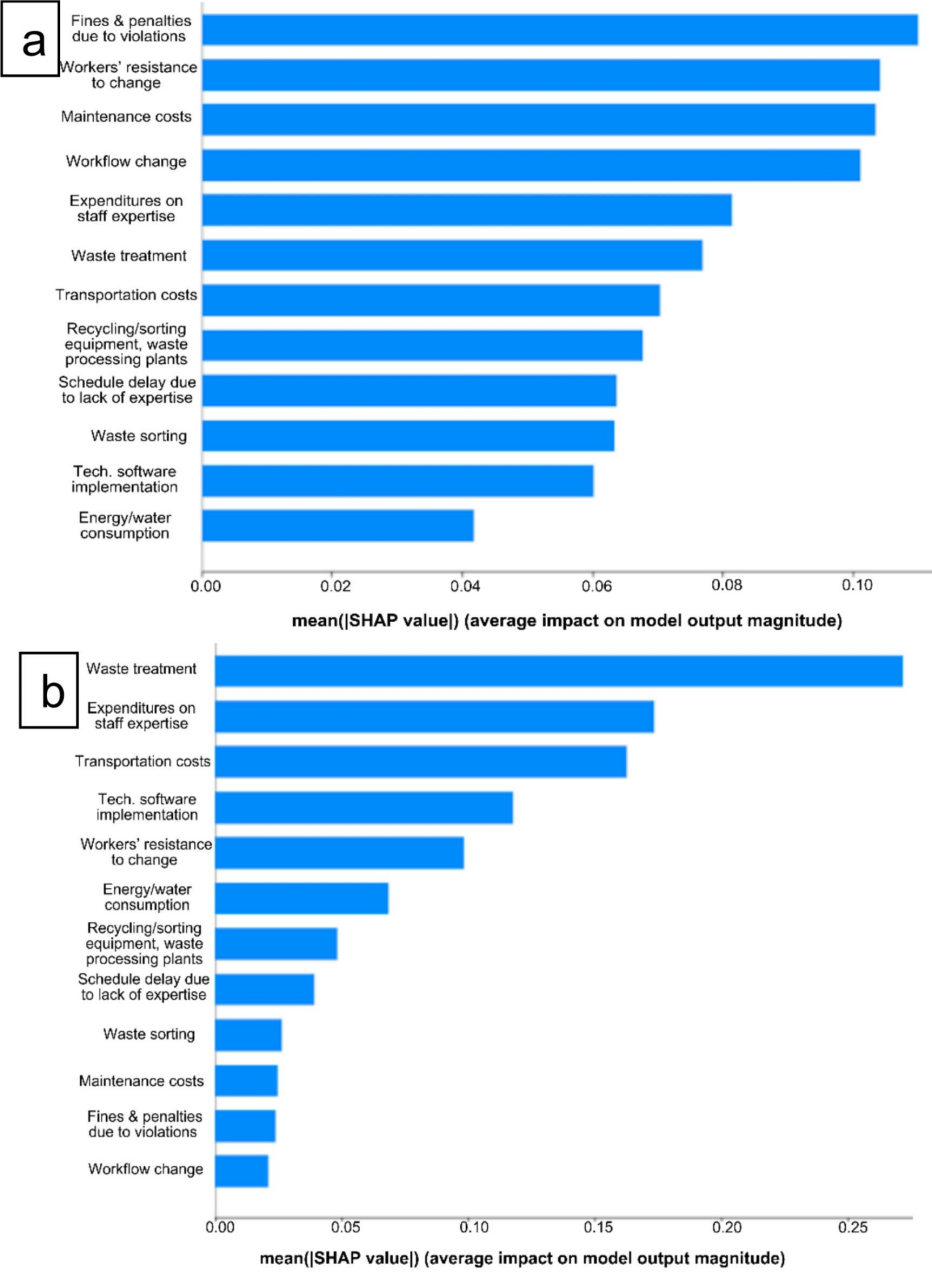
SHAP values plot from top to bottom (a) European, MAPE: 29.89%, (b) non-European, MAPE: 33.10%. Source: Authors.

This model linked the costliness of CE material efficiency strategies (average score of the sub-questions in Question 10) with the perceived costs of key enablers and drivers for these strategies (Question 12.1–12.12). Figure 6 illustrates the results for the studied (a) European and (b) non-European countries. Within the studied European countries, results indicate that regulatory non-compliance, resulting in fines and penalties, is a top cost driver. Additionally, reduced work efficiency, stemming from workers’ resistance to change, significantly impacts overall expenses, compounded by maintenance costs and workflow disruptions due to necessary adjustments. The data also points to concerns about expenditures related to staff expertise, reflecting the costs associated with training and development.

In contrast, for the studied non-European countries, waste treatment costs stand out, potentially indicating less-developed waste management infrastructure. The shared costs associated with transportation, technological upgrades, and staff expertise between the studied European and non-European datasets illustrate these as potential global challenges for CE implementation. Notably, both groups of countries cite work efficiency as being affected by resistance to change, which speaks to a widespread challenge in managing human factors in organizational change.

The findings in Fig. 6a indicate that non-compliance costs are a primary concern within the studied European countries. A similar emphasis on regulatory penalties tied to sustainable C&DW treatment has been noted elsewhere^35^, likely reflecting the stringent regulatory environment in Europe (e.g., 70% of waste should be reused, recycled, or recovered)^32^. Reduced work efficiency due to resistance to change is also a significant cost driver, which can be explained by the higher labor costs in European countries^36^. Labor costs are claimed to be among the most critical barriers to C&DW recycling in Europe^34^. This resistance is linked to a lack of awareness and interest, also listed as a significant barrier in Fig. 6a, which is aligned with other studies^13,36^. Maintenance costs, identified as a top contributor to financial expenses^15^, can disrupt established procedures and lead to additional costs.

On the other hand, while waste sorting was considered less important among the participants of this research, it still contributes to the overall cost of C&DW management. However, in a more recent study^34^, European stakeholders identified waste sorting as a significant factor in C&DW management. Thus, while waste sorting may have been previously considered a less significant contributor to financial expenses, it might be gaining more attention as a crucial aspect of C&DW management in the European context.

In the studied non-European countries (Fig. 6b), the significance of waste treatment costs is noteworthy, potentially indicating less developed waste management infrastructure, as suggested by other scholars^37,38^. The importance of proper waste treatment using various techniques in developing countries is also emphasized^39^. In Kazakhstan, for example, the cost of managing waste does not significantly impact the amount of waste that is reused and recycled^40^. The results provided in Fig. 6b also highlight stakeholders’ concerns regarding expenditures related to staff expertise, reflecting the costs associated with training and development, which was underscored in^29^. Similarly, Turkish scholars emphasized the need for appropriate expertise among local construction workers^41^. A unique situation is observed in the UAE, where the prevalence of short-term contracts leads to a continuous workflow and the need for repeated training for new employees. This situation hampers the overall development of a stable work culture^29^.

Another significant concern identified is the cost of transportation (Fig. 6b). Stakeholders in Kazakhstan’s construction sector deemed the transportation costs of C&DW to be very important^7^. This emphasizes the need for efficient logistics and transportation strategies to manage construction waste. Fines and penalties in non-European countries received low scores in Fig. 6b, probably because they might be lower or less stringent than in Europe.

Considering the results from the studied European countries in our survey, we suggest following a balanced approach to non-compliance costs, raising awareness and motivation among workers to avoid their resistance to change, and carefully planning for possible maintenance costs. Meanwhile, in the studied non-European countries, the development of waste treatment infrastructure, qualitative employee training, and considerate planning of the most efficient logistics could play an essential role in alleviating the potential economic burdens associated with CE material efficiency strategies. The results also show that the understanding of CE differs between European and non-European countries; for instance, maintenance costs are much more critical for European stakeholders. The broader range of mean values for non-European countries compared to European ones regarding cost escalation factors is also noteworthy and suggests clearer paths for improving CE implementation.

#### Benefits

**Fig. 7:**
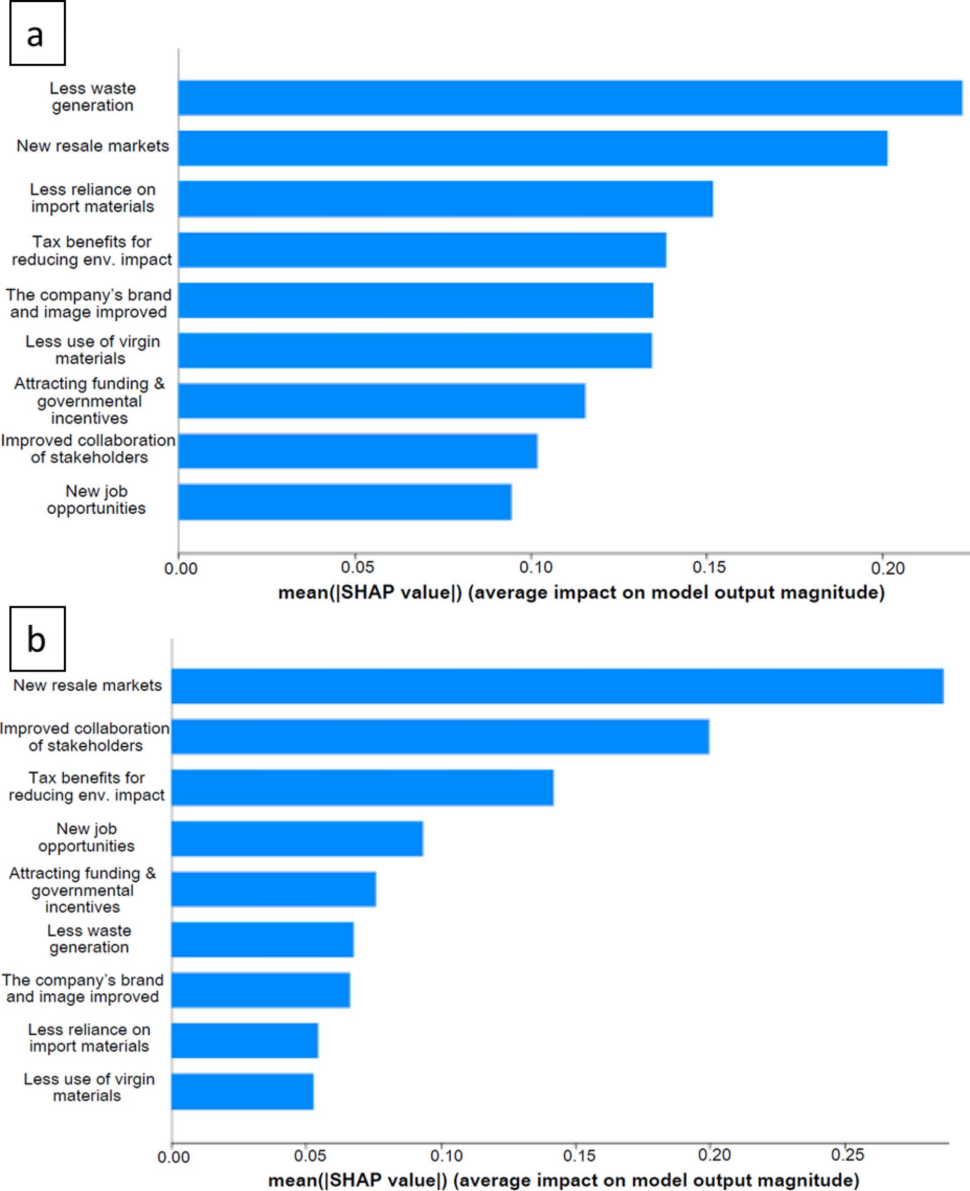
SHAP values plot from top to bottom (a) European, MAPE: 32.55%, (b) non-European, MAPE: 17.75%. Source: Authors.

The final model (Fig. 7) links the level of specific benefits of CE material efficiency strategies (average score of the sub-questions in Question 9) with stakeholders’ perceptions of where these benefits originate (Question 11.1–11.9). The survey responses highlight differing significance among various sources of specific benefits between the two groups of countries. Among the studied European countries, there is a marked emphasis on advantages from reducing waste, alongside benefits from using local materials and an improved public image of CE strategies. In contrast, the data from the studied non-European countries reveals different results, emphasizing the potential for market developments as an important benefit, focusing on features like establishing resale opportunities, encouraging partnerships among construction parties, and exploring potential tax advantages.

In the responses from the studied European countries (see Fig. 7a), a greater focus is placed on waste reduction, which resonates with previous findings where the reuse of materials was a top contributor to both costs and benefits. The results in Fig. 7a demonstrate Europe’s strong commitment to decreasing waste and enhancing reuse, aligning with legislative acts and national strategies. Additionally, new resale markets emerge as the second most impactful factor on specific benefits, indicating a growing economic landscape where CE is becoming a key driver. This interest in creating markets for used, upcycled, recycled, and repurposed goods supports environmental goals and fosters new business opportunities. Indeed, construction and demolition waste reuse and recycling markets were predicted to be beneficial, provided that solid stakeholder collaboration is ensured^42^. For example, a study in Spain concluded that although the pursuit of construction sustainability has driven the use of partially or wholly waste-based eco-materials, current business models need revision for this to be successful^43^.

The results in Fig. 7b for the studied non-European countries show that greater emphasis is placed on factors such as new resale markets, improved stakeholder collaboration, and potential financing attracted through reduced environmental impact. These factors emphasize the creation of new channels for business expansion and income generation. The interest of Kazakhstani stakeholders in resale markets was also presented in previous studies^6,7^. While there is a consensus between the studied European and non-European countries on the importance of developing new markets, there seems to be less enthusiasm for reducing waste generation in non-European countries. This discrepancy could stem from several factors, including economic considerations, existing waste management infrastructure, and the perceived immediacy of economic benefits versus long-term environmental benefits.

Notably, prioritizing new market development over waste reduction highlights a potential area for policy refinement and awareness-raising to ensure a balanced approach to economic and circular economy development. Both regional contexts are highly motivated to benefit from waste reuse and its resale markets. The European focus on waste reduction is closely aligned with national legislation and strategies, demonstrating a solid commitment to environmental sustainability. The region has invested significantly in recycling infrastructure and technology, reinforcing its commitment to reusing materials. In contrast, non-European countries exhibit a slightly different approach, with greater emphasis on collaborative construction efforts and funding opportunities due to reduced environmental impact. The results highlight the importance of adapting waste management strategies to each region’s specific circumstances and priorities while encouraging greater global coordination and cooperation to address common challenges in waste generation and management.

## Conclusions and future implications

The primary objective of this study was to assess the perceived costs and benefits of CE material efficiency strategies from the perspectives of diverse stakeholders in the construction industry. We used a machine learning model (XGBoost) to analyze the survey data, identifying important factors and patterns in responses. To interpret the model’s predictions, we used SHAP values, which allowed us to see the impact of each survey question on the results, giving us a view of which factors were most influential. Our findings provide valuable recommendations for policymakers, businesses, and other stakeholders seeking to implement CE methods by revealing nuanced differences and commonalities in stakeholder perceptions across European and non-European contexts.

The results reveal distinct regional variations between the European and non-European countries studied in terms of their perception of the costs and benefits of various CE material efficiency strategies. In Europe, strategies such as optimizing the reuse of materials, utilizing disassembly elements, and offsite production are recognized as reducing costs while also delivering closely aligned benefits of material reuse, offsite production, and recycling. This demonstrates the dual value of these strategies in both minimizing expenses and maximizing benefits. Similarly, in non-European countries, the emphasis on offsite production and material reuse reflects their recognition as strategies that can reduce costs while also being beneficial, further highlighting the global relevance of these approaches. However, the two regions also face unique challenges, such as waste treatment costs, which may reflect differences in infrastructural capacity. The analysis also identifies shared challenges across the EU and non-EU regions, particularly in managing human factors in CE implementation, such as resistance to change, which impacts work efficiency and ultimately affects both costs and benefits.

Overall, the analysis provides valuable insights for planning and decision-making in CE implementation. For instance, the consistent recognition of material reuse and offsite production as crucial factors across both European and non-European contexts suggests that these strategies offer robust opportunities for balancing costs and benefits. Meanwhile, region-specific concerns, such as waste management and market development in non-European countries, highlight areas where tailored approaches may be needed to support CE implementation. By identifying potential risks and opportunities and considering stakeholder interests, this analysis equips governments and funding institutions to refine regulations and create or adapt incentives. supporting the implementation of circular economy principles in the construction sector.

While this study provides valuable insights into the economic impacts of CE implementation, it is important to acknowledge limitations related to the sample’s representativeness. The research included stakeholders from both EU and non-EU countries to capture a diverse range of perspectives on CE practices. However, the sample may not fully represent the broader landscape of EU and non-EU countries due to inherent variability within these regions. Additionally, while XGBoost and SHAP provide powerful insights, their application to survey data presents challenges in generalizability and reliability, partly due to the data split and separate modelling for EU and non-EU subsets. This approach may limit the models’ ability to capture nuanced regional differences and could affect the reproducibility of results.

## Electronic supplementary material

Below is the link to the electronic supplementary material.

Supplementary Material 1

## Data Availability

Data is available by the following link: https://github.com/aidanatleuken/cost_benefit_CE.
