# Supplementary material for: Stakeholder perspectives on the costs and benefits of circular construction
Source: Sci Rep. 2024 Dec 3;14:30039. doi: 10.1038/s41598-024-81741-z (PMC11614893; doi:10.1038/s41598-024-81741-z)
Supplement: Supplementary file 1 — Supplementary Material 1 [file 41598_2024_81741_MOESM1_ESM.docx]

## Supplementary file for the manuscript titled “Stakeholder Perspectives on the Costs and Benefits of Circular Construction”

## Supplementary Information I. Survey questions

Q1 Working country

▼ Afghanistan (1) ... Zimbabwe (1357)

Q2. Construction industry experience?

- 1-5 years (1)
- 6-10 years (2)
- 11-15 years (3)
- 15-20 years (4)
- over 20 years (5)

Q3. How many employees are there working in your company/ organization / institution?:

- less than 10 (1)
- 11-50 (2)
- 50-100 (3)
- 101-500 (4)
- 501-1000 (5)
- more than 1000 (6)

Q4. Type of stakeholder that you represent the best

- Academician/Researcher (13)
- Client and/or Investor (1)
- Project manager (2)
- Material supplier (3)
- Manufacturer (4)
- Technician and/or Engineer (5)
- Designer Architect and/or Engineer (6)
- Contractor (7)
- End-user (8)
- Government and/or Councillor (9)
- Urban designer (10)
- Environmental agency (11)
- Other, please specify (12) __________________________________________________

Q5. Has your company/ organization / institution (or you) been involved in green /sustainable/circular building practices?

- Yes (4)
- No (5)
- Not sure (6)

Q6. Please describe a case when your company/ organization / institution used circular economy practices. Elaborate on the perceived costs and benefits for your organization.

________________________________________________________________

**End of Block: Personal information**

**Start of Block: Identifying stakeholder’s interests and relevance**

*Display This Question: If Type of stakeholder that you represent the best = Academician/Researcher*

Q7. Please answer the questions given below on circular economy practices related to construction materials

|  | Very low (1) | Low (2) | Moderate (3) | High (4) | Very high (5) |
| --- | --- | --- | --- | --- | --- |
| How important are the benefits of circular economy practices for your organization? (1) |  |  |  |  |  |
| How important are the costs of circular economy practices for your organization? (2) |  |  |  |  |  |
| How do you think circular economy can impact your organization’s financial performance? (3) |  |  |  |  |  |
| How do you think circular economy can impact your organization’s reputation and brand image? (4) |  |  |  |  |  |

**End of Block: Identifying stakeholder’s interests and relevance**

**Start of Block: Determination of costs and benefits**

*Display This Question: If Type of stakeholder that you represent the best = Academician/Researcher*

Q8. Please indicate your level of agreement with the following statements

|  |  |
| --- | --- |
|  |  |
| My company optimizes the number of structural elements used (1) | ▼ Fully disagree (1 ... Fully agree (5) |
| My company uses structure elements that can be easily disassembled (2) | ▼ Fully disagree (1 ... Fully agree (5) |
| My company produces structure elements offsite (3) | ▼ Fully disagree (1 ... Fully agree (5) |
| My company maximizes storing construction materials for reuse (4) | ▼ Fully disagree (1 ... Fully agree (5) |
| My company optimizes the reuse of construction materials (5) | ▼ Fully disagree (1 ... Fully agree (5) |
| My company sells or exchanges used construction materials (6) | ▼ Fully disagree (1 ... Fully agree (5) |
| My company recycles construction materials (7) | ▼ Fully disagree (1 ... Fully agree (5) |

| Page Break |  |
| --- | --- |

Q9. Please indicate your level of agreement with the following statements

|  |  |
| --- | --- |
|  |  |
| Optimizing the number of structural elements used provides benefits (1) | ▼ Fully disagree (1 ... Fully agree (5) |
| Using structure elements that can be easily disassembled provides benefits (2) | ▼ Fully disagree (1 ... Fully agree (5) |
| Producing structure elements offsite provides benefits (3) | ▼ Fully disagree (1 ... Fully agree (5) |
| Maximizing storing construction materials for reuse provides benefits (4) | ▼ Fully disagree (1 ... Fully agree (5) |
| Optimizing the reuse of construction materials provides benefits (5) | ▼ Fully disagree (1 ... Fully agree (5) |
| Selling or exchanging used construction materials provides benefits (6) | ▼ Fully disagree (1 ... Fully agree (5) |
| Recycling construction materials provides benefits (7) | ▼ Fully disagree (1 ... Fully agree (5) |

| Page Break |  |
| --- | --- |

Q10. Please indicate your level of agreement with the following statements

|  |  |
| --- | --- |
|  |  |
| Optimizing the number of structural elements used is costly (1) | ▼ Fully disagree (1 ... Fully agree (5) |
| Using structure elements that can be easily disassembled is costly (2) | ▼ Fully disagree (1 ... Fully agree (5) |
| Production of structure elements offsite is costly (3) | ▼ Fully disagree (1 ... Fully agree (5) |
| Maximizing storing construction materials for reuse is costly (4) | ▼ Fully disagree (1 ... Fully agree (5) |
| Optimizing the reuse of construction materials is costly (5) | ▼ Fully disagree (1 ... Fully agree (5) |
| Selling or exchanging used construction materials is costly (6) | ▼ Fully disagree (1 ... Fully agree (5) |
| Recycling construction materials is costly (7) | ▼ Fully disagree (1 ... Fully agree (5) |

**End of Block: Determination of costs and benefits**

**Start of Block: Analyzing, reflecting, and weighting expenses and revenues**

*Display This Question: If Type of stakeholder that you represent the best = Academician/Researcher*

Q11. How would your organization benefit in terms of cost reductions from circular economy practices (listed in the previous table) that focus on materials? Please rate their potential benefits and cost savings on a scale of 1 (minimal) to 5 (maximal) or N/A if the practice is not available in your company

|  |  |
| --- | --- |
| Less waste generation (1) | ▼ 1 (1) ... NA (6) |
| Less use of virgin materials (2) | ▼ 1 (1) ... NA (6) |
| New resale markets (3) | ▼ 1 (1) ... NA (6) |
| Less reliance on import materials (4) | ▼ 1 (1) ... NA (6) |
| New job opportunities (5) | ▼ 1 (1) ... NA (6) |
| Tax benefits for reducing environmental impact (6) | ▼ 1 (1) ... NA (6) |
| Improved collaboration among stakeholders (7) | ▼ 1 (1) ... NA (6) |
| The company’s brand and image improved (8) | ▼ 1 (1) ... NA (6) |
| Attracting potential funding and governmental incentives (9) | ▼ 1 (1) ... NA (6) |

*Display This Question: If Type of stakeholder that you represent the best = Academician/Researcher*

Q12. Below is a list of actions that may increase the costs of CE implementations. Please rate their potential cost increase on a scale of 1 to 5 or NA if the practice is not available in your company

|  |  |
| --- | --- |
| Waste sorting (1) | ▼ 1 (1) ... NA (6) |
| Waste treatment (2) | ▼ 1 (1) ... NA (6) |
| Recycling/sorting equipment, waste processing plants, and related technologies (3) | ▼ 1 (1) ... NA (6) |
| Energy and water consumption for aggregate cleaning (4) | ▼ 1 (1) ... NA (6) |
| Transportation costs in general (5) | ▼ 1 (1) ... NA (6) |
| Maintenance costs (6) | ▼ 1 (1) ... NA (6) |
| Technological software implementation (7) | ▼ 1 (1) ... NA (6) |
| Expenditures on staff expertise (8) | ▼ 1 (1) ... NA (6) |
| Workflow change (9) | ▼ 1 (1) ... NA (6) |
| Schedule delay due to lack of expertise (10) | ▼ 1 (1) ... NA (6) |
| Work efficiency is reduced due to workers’ resistance to change (11) | ▼ 1 (1) ... NA (6) |
| Violations resulting in fines & penalties (12) | ▼ 1 (1) ... NA (6) |

**End of Block: Analyzing, reflecting, and weighting expenses and revenues**

**Start of Block: Block 5**

*Display This Question: If Type of stakeholder that you represent the best = Academician/Researcher*

Q13. Please evaluate the projected **costs** in monetary terms in comparison to standard practices, with particular emphasis on the phases of a typical construction project

|  | Planning, Design, Procurement | Building Construction | Building Certification | Building Operation and Maintenance | Demolition |
| --- | --- | --- | --- | --- | --- |
|  |  |  |  |  |  |
| Optimize the number of structural elements used (2) | ▼ Very low (1 ... Very high (5) | ▼ Very low (1 ... Very high (5) | ▼ Very low (1 ... Very high (5) | ▼ Very low (1 ... Very high (5) | ▼ Very low (1 ... Very high (5) |
| Use structure elements that can be easily disassembled (3) | ▼ Very low (1 ... Very high (5) | ▼ Very low (1 ... Very high (5) | ▼ Very low (1 ... Very high (5) | ▼ Very low (1 ... Very high (5) | ▼ Very low (1 ... Very high (5) |
| Produce structure elements offsite (4) | ▼ Very low (1 ... Very high (5) | ▼ Very low (1 ... Very high (5) | ▼ Very low (1 ... Very high (5) | ▼ Very low (1 ... Very high (5) | ▼ Very low (1 ... Very high (5) |
| Store construction materials for reuse (5) | ▼ Very low (1 ... Very high (5) | ▼ Very low (1 ... Very high (5) | ▼ Very low (1 ... Very high (5) | ▼ Very low (1 ... Very high (5) | ▼ Very low (1 ... Very high (5) |
| Reuse construction materials (6) | ▼ Very low (1 ... Very high (5) | ▼ Very low (1 ... Very high (5) | ▼ Very low (1 ... Very high (5) | ▼ Very low (1 ... Very high (5) | ▼ Very low (1 ... Very high (5) |
| Sell or exchange used construction materials (7) | ▼ Very low (1 ... Very high (5) | ▼ Very low (1 ... Very high (5) | ▼ Very low (1 ... Very high (5) | ▼ Very low (1 ... Very high (5) | ▼ Very low (1 ... Very high (5) |
| Recycle construction materials (8) | ▼ Very low (1 ... Very high (5) | ▼ Very low (1 ... Very high (5) | ▼ Very low (1 ... Very high (5) | ▼ Very low (1 ... Very high (5) | ▼ Very low (1 ... Very high (5) |

| Page Break |  |
| --- | --- |

*Display This Question: If Type of stakeholder that you represent the best = Academician/Researcher*

Q14. Please evaluate the projected **benefits** in monetary terms in comparison to standard practices, with particular emphasis on the phases of a typical construction project

|  | Planning, Design, Procurement | Building Construction | Building Certification | Building Operation and Maintenance | Demolition |
| --- | --- | --- | --- | --- | --- |
|  |  |  |  |  |  |
| Optimize the number of structural elements used (2) | ▼ Very low (1 ... Very high (5) | ▼ Very low (1 ... Very high (5) | ▼ Very low (1 ... Very high (5) | ▼ Very low (1 ... Very high (5) | ▼ Very low (1 ... Very high (5) |
| Use structure elements that can be easily disassembled (3) | ▼ Very low (1 ... Very high (5) | ▼ Very low (1 ... Very high (5) | ▼ Very low (1 ... Very high (5) | ▼ Very low (1 ... Very high (5) | ▼ Very low (1 ... Very high (5) |
| Produce structure elements offsite (4) | ▼ Very low (1 ... Very high (5) | ▼ Very low (1 ... Very high (5) | ▼ Very low (1 ... Very high (5) | ▼ Very low (1 ... Very high (5) | ▼ Very low (1 ... Very high (5) |
| Store construction materials for reuse (5) | ▼ Very low (1 ... Very high (5) | ▼ Very low (1 ... Very high (5) | ▼ Very low (1 ... Very high (5) | ▼ Very low (1 ... Very high (5) | ▼ Very low (1 ... Very high (5) |
| Reuse construction materials (6) | ▼ Very low (1 ... Very high (5) | ▼ Very low (1 ... Very high (5) | ▼ Very low (1 ... Very high (5) | ▼ Very low (1 ... Very high (5) | ▼ Very low (1 ... Very high (5) |
| Sell or exchange used construction materials (7) | ▼ Very low (1 ... Very high (5) | ▼ Very low (1 ... Very high (5) | ▼ Very low (1 ... Very high (5) | ▼ Very low (1 ... Very high (5) | ▼ Very low (1 ... Very high (5) |
| Recycle construction materials (8) | ▼ Very low (1 ... Very high (5) | ▼ Very low (1 ... Very high (5) | ▼ Very low (1 ... Very high (5) | ▼ Very low (1 ... Very high (5) | ▼ Very low (1 ... Very high (5) |

**End of Block: Block 5**

**Start of Block: Block 8**

Q15. If you want to review and potentially change your previous response to the question
"Please describe a case when your company used circular economy practices. Elaborate on the perceived costs and benefits for your organization."
If you have nothing to add, please proceed 

Supplementary Information II. Survey Development


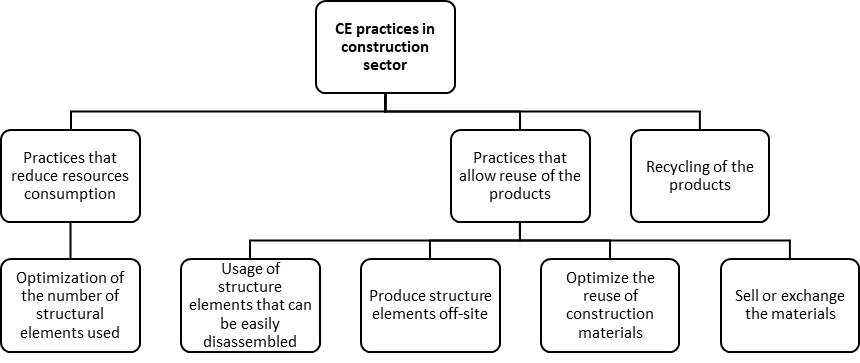


**Figure S1.** Circular Economy practices in the construction sector related to materials

Source: Authors.

Benefits

Less waste generation

Less use of virgin materials

New resale markets

Less reliance on import materials

New job opportunities

Tax benefits

Improved collaboration among stakeholders

The company’s brand and image improved

Attracting potential funding

Costs

Waste sorting

Waste treatment

Recycling/sorting equipment, etc

Resources consumption for aggregate cleaning

Transportation costs in general

Maintenance costs

Technological software

Expenditures on staff expertise

Workflow change

Schedule delay due to lack of expertise

Workers’ resistance to change

Violations ending up with fines/penalties

**Figure S2. Circular Economy implementation costs and benefits (listed in the questionnaire (Q11 and Q 12))**

Source: Authors.

Supplementary Information III. Results of XGBoost and SHAP modelling based on the total dataset

The first model was developed by correlating the perceived importance of costs linked to circular economy practices (Question 7.2) with the costliness of CE material efficiency strategies (Questions 10.1–10.7). Figure S3 shows the obtained SHAP values.


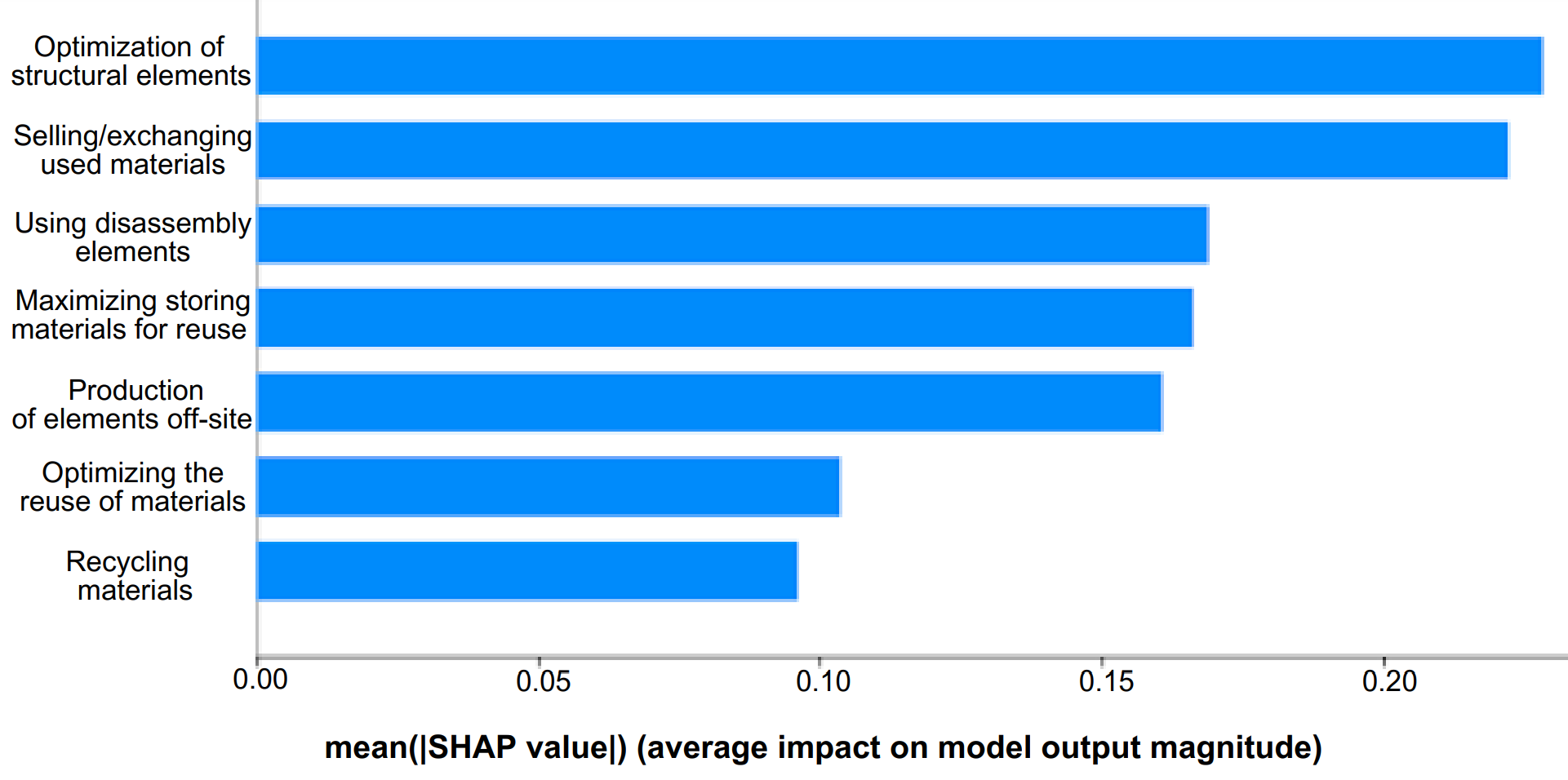


**Figure S3. SHAP values plot, MAPE: 29.65%**

The second model has linked the costliness of material efficiency strategies (average score of the sub-questions in Question 10) with the perception of the costs attached to key enablers/drivers of such strategies (Question 12.1–12.12). Figure S4 shows the obtained SHAP values.


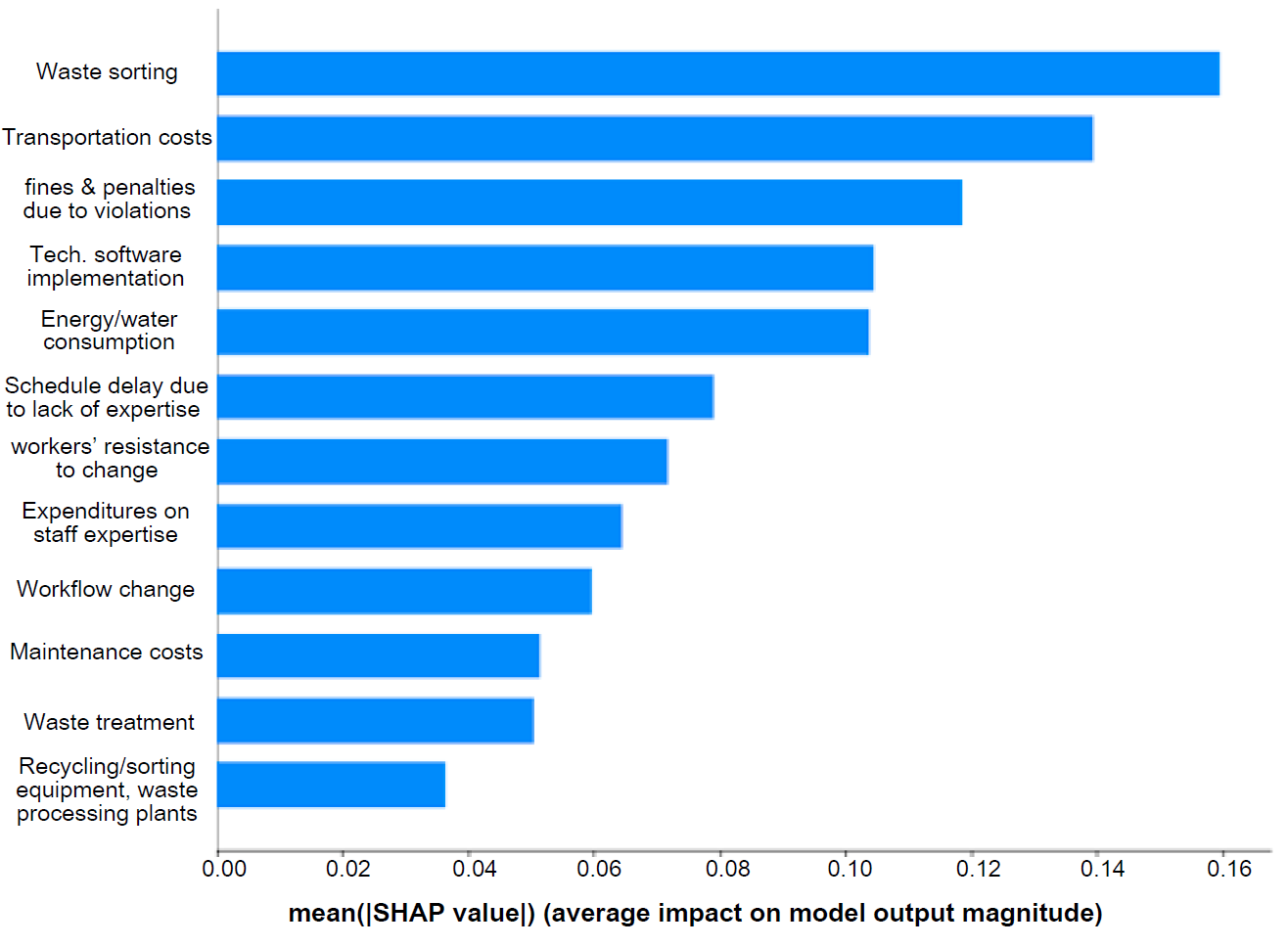


**Figure S4. SHAP values plot, MAPE: 21.40%**

The third model focused on assessing the importance of benefits associated with circular economy (CE) material efficiency strategies. The model linked the responses to Question 7.1, which asked "How important are the benefits of circular economy practices for your organization?", with the level of agreement on the benefits of common CE material efficiency strategies (Question 9.1–9.7). Figure S5 shows the obtained SHAP values.


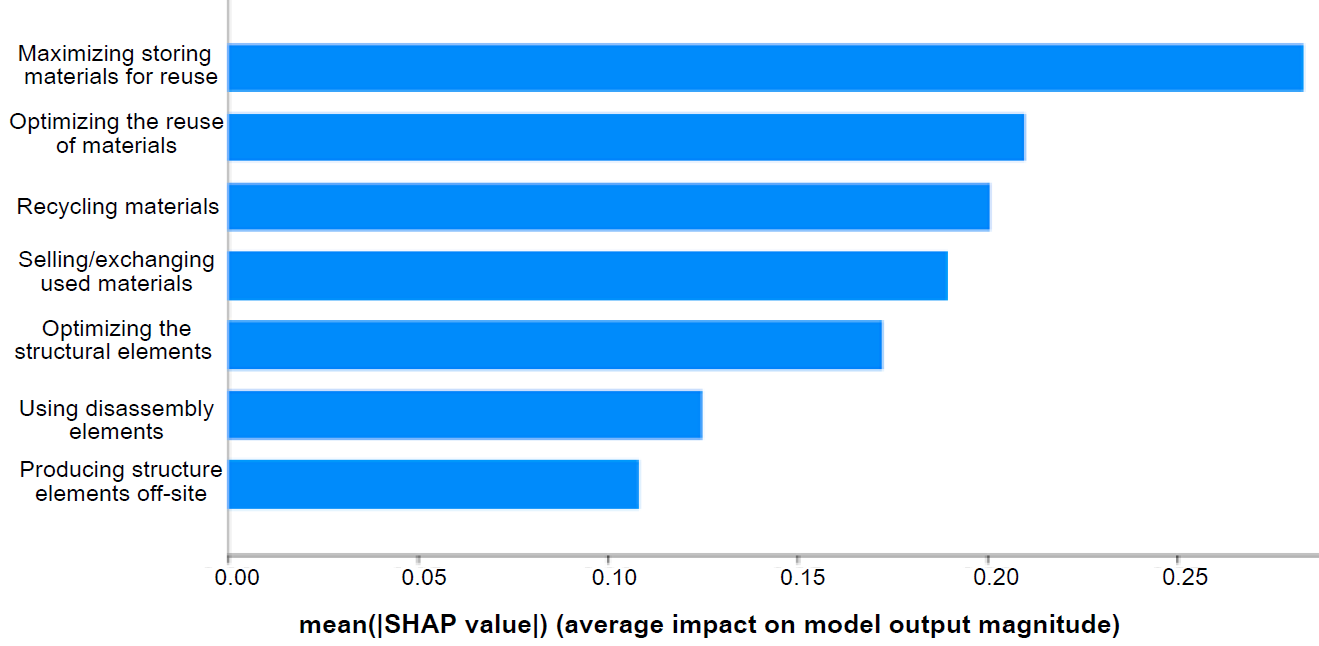


**Figure S5. SHAP values plot, MAPE:32.27%**

The fourth model linked the level of benefits of CE material efficiency strategies (average score of the sub-questions in Question 9) with the stakeholders’ perception on where these benefits are coming from (Question 11.1–11.9). Figure S6 shows the obtained SHAP values.


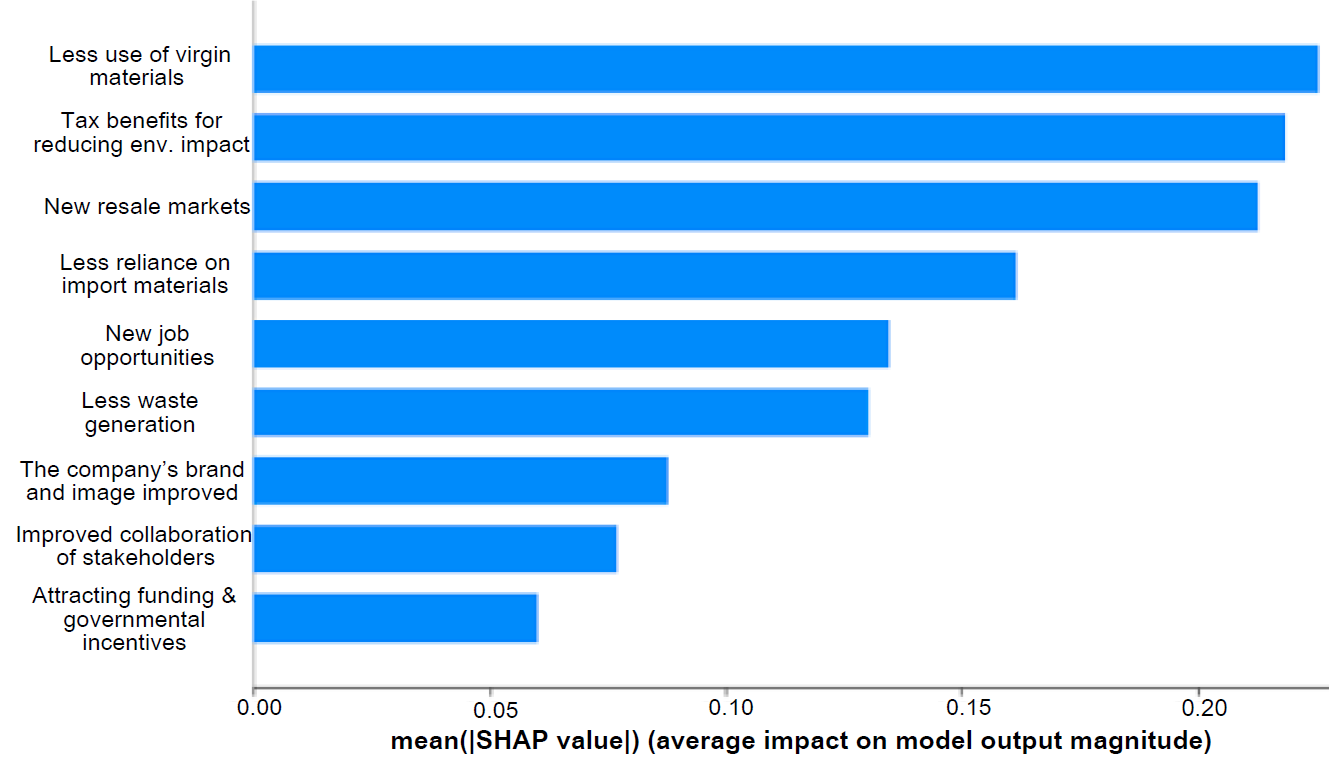


**Figure S6. SHAP values plot, MAPE: 24.50%**
